# Ultrasound attenuation of cortical bone correlates with biomechanical, microstructural, and compositional properties

**DOI:** 10.1186/s41747-023-00418-w

**Published:** 2024-02-06

**Authors:** Saeed Jerban, Victor Barrere, Behnam Namiranian, Yuanshan Wu, Salem Alenezi, Erik Dorthe, Darryl Dlima, Sameer B. Shah, Christine B. Chung, Jiang Du, Michael P. Andre, Eric Y. Chang

**Affiliations:** 1 Department of Radiology, University of California, San Diego, 9500 Gilman Dr, La Jolla, CA 92093 USA; 2 Research Service, Veterans Affairs San Diego Healthcare System, 3350 La Jolla Village Drive, San Diego, CA 92161 USA; 3 Department of Orthopaedic Surgery, University of California, San Diego, La Jolla, CA USA; 4 Research and Laboratories Sector, Saudi Food and Drug Authority, Riyadh, Kingdom of Saudi Arabia; 5 Shiley Center for Orthopedic Research and Education at Scripps Clinic, La Jolla, CA USA

**Keywords:** Bone mechanics, Cortical bone, MRI, Quantitative ultrasound, Ultrashort echo time

## Abstract

**Background:**

We investigated the relationship of two commonly used quantitative ultrasound (QUS) parameters, speed of sound (SoS) and attenuation coefficient (*α*), with water and macromolecular contents of bovine cortical bone strips as measured with ultrashort echo time (UTE) magnetic resonance imaging (MRI).

**Methods:**

SoS and *α* were measured in 36 bovine cortical bone strips utilizing a single-element transducer with nominal 5 MHz center frequency based on the time of flight principles after accommodating for reflection losses. Specimens were then scanned using UTE MRI to measure total, bound, and pore water proton density (TWPD, BWPD, and PWPD) as well as macromolecular proton fraction and macromolecular transverse relaxation time (T2-MM). Specimens were also scanned using microcomputed tomography (μCT) at 9-μm isometric voxel size to measure bone mineral density (BMD), porosity, and pore size. The elastic modulus (E) of each specimen was measured using a 4-point bending test.

**Results:**

*α* demonstrated significant positive Spearman correlations with E (*R* = 0.69) and BMD (*R* = 0.44) while showing significant negative correlations with porosity (*R* = -0.41), T2-MM (*R* = -0.47), TWPD (*R* = -0.68), BWPD (*R* = -0.67), and PWPD (*R* = -0.45).

**Conclusions:**

The negative correlation between *α* and T2-MM is likely indicating the relationship between QUS and collagen matrix organization. The higher correlations of *α* with BWPD than with PWPD may indicate that water organized in finer structure (bound to matrix) provides lower acoustic impedance than water in larger pores, which is yet to be investigated thoroughly.

**Relevance statement:**

This study highlights the importance of future investigations exploring the relationship between QUS measures and all major components of the bone, including the collagenous matrix and water. Investigating the full potential of QUS and its validation facilitates a more affordable and accessible tool for bone health monitoring in clinics.

**Key points:**

• Ultrasound attenuation demonstrated significant positive correlations with bone mechanics and mineral density.

• Ultrasound attenuation demonstrated significant negative correlations with porosity and bone water contents.

• This study highlights the importance of future investigations exploring the relationship between QUS measures and all major components of the bone.

**Graphical Abstract:**

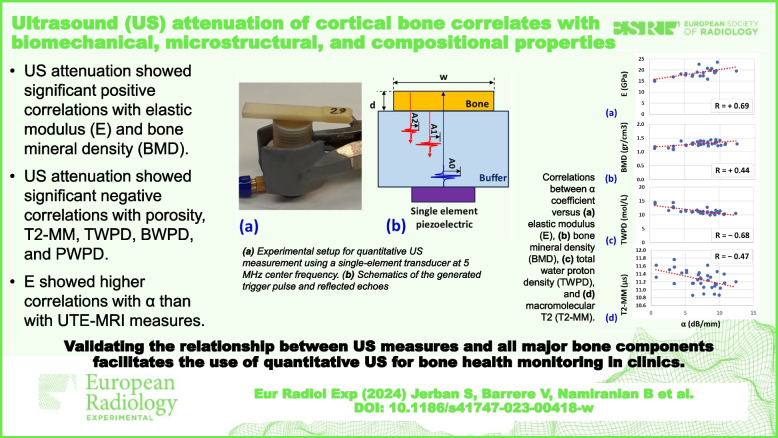

## Background

Cortical bone plays an integral role in bone resistance to fracture [1, 2]. Therefore, an accurate evaluation of cortical bone in critical sites, such as the proximal femur, may improve diagnosis and treatment monitoring in patients with primary or secondary osteoporosis. Cortical bone is mainly comprised of a mineral matrix (~40% by volume), an organic matrix (~30%), water (~20%), and fat (< 5%) [3, 4]. In healthy bone, most of the water is bound to the organic and mineral matrices, called “bound water” (BW) [5–11]. A smaller portion of bone water, called “pore water” (PW), resides in different pores such as Haversian canals (10–200 μm), lacunae (1–10 μm), and canaliculi (0.1–1 μm) [3, 5].

Bone mineral density (BMD), as measured by dual-energy x-ray absorptiometry (DXA) at the spine or hip, is the standard clinical measure to diagnose osteoporosis and estimate bone fracture risk [12–15]. Despite the widespread use of BMD in clinics, a diagnosis of osteoporosis (based on DXA *T*-score of -2.5 or less) often fails to predict fracture risk accurately [16–23]. Notably, the DXA-based BMD measurement cannot detect accurate local changes in bone structure due to its two-dimensional nature. Notably, the recently developed trabecular bone score [24] and bone strain index [25] provide a localized bone assessment by employing textural and morphological processing of the DXA images; however, they cannot detect three-dimensional bone changes. Moreover, all x-ray-based bone assessment techniques focus on the mineral component of the bone while missing the other critical components, such as collagenous matrix and water.

Magnetic resonance imaging (MRI) has been increasingly used for cortical bone assessment [26–28], first to avoid exposure to ionizing radiation associated with x-ray-based techniques and second to provide an opportunity for simultaneous evaluation of the surrounding soft tissues [29]. Notably, clinical MRI is not able to detect a considerable signal from cortical bone due to its short apparent transverse relaxation time (T2* ≈ 0.4 ms). However, ultrashort echo time (UTE) MRI can image cortical bone, consequently enabling quantitative assessment of cortical bone [11, 26–28, 30–34]. Typically, UTE MRI techniques can acquire the bone signal in less than 50 μs after radiofrequency excitation and before a significant decay in transverse magnetization. Quantitative UTE MRI has been reported to evaluate water contents (*i.e.*, PW, BW, and total water [TW]) and macromolecular proton fraction (MMF) in cortical bone using a combination of basic UTE, inversion recovery UTE, and magnetization transfer UTE (UTE-MT) modeling [34, 35]. Nevertheless, UTE-MRI-based evaluation of bone is underutilized partly due to the high cost and time demands of MRI in general.

Quantitative ultrasound (QUS) techniques have also been developed to assess cortical bone, motivated by the need to provide portable, easily accessible, and affordable techniques for osteoporosis assessment without exposure to ionizing radiation [36, 37]. QUS-based parameters for bone assessment have been mainly focused on estimating the ultrasound (US) wave velocity (or speed of sound [SoS]), US attenuation, normalized US attenuation over frequency ranges, and US backscatter [38]. Such measurements can be performed through US pulse-echo [39, 40], transverse transmission [41], or axial transmission [42, 43] techniques targeting different skeletal sites such as the finger, spine, hip, femur, tibia, and heel [37, 39–49]. The main perspective of these studies has been the search for a QUS measure with a significant correlation with BMD [37, 42, 43, 45, 47, 50].

Most of the reported QUS studies missed exploring the relationship with other major components of the cortical bone, such as water and macromolecular contents that comprise up to 60% of the bone volume [3, 4, 51, 52].

The goal of this study was to investigate the relationship of two widely used QUS measures, SoS and attenuation coefficient (*α*), with water and macromolecular contents of bovine cortical bone strips as measured with UTE-MRI-based methods. We also aimed to explore correlations of SoS and *α* with microstructural and mechanical properties of bone strips.

## Methods

### Sample preparation

Four fresh bovine femoral midshafts were obtained, and the central portions of the shafts were cut into 40-mm segments using a commercial band saw (B16, Butcher Boy, TN, USA). In total, 36 rectangular bone strips were excised from the bone shafts using a low-speed diamond saw (Isomet 1000, Buehler, IL, USA). The final dimensions of the rectangular bone strips were approximately 6 × 3 × 40 mm. The 3-mm dimension of the specimens was in the radial direction of the original femoral shafts. Bone strips were immersed in phosphate-buffered saline for one hour at room temperature before each scanning process described in the following sections.

### Quantitative ultrasound

A single-element US transducer (ULTRAN, State College, PA, USA) with a 2.3-MHz center frequency (5 MHz nominal center frequency, half bandwidth of 1.4–4.1 MHz) was used to measure SoS and *α* coefficients along the ~3 mm thickness of the bone strips. A commercial pulser/amplifier (model 5052PR, Panametrics, MA, USA) was used to trigger the transducer pulses, connected to the transducer by a waterproof BNC (Bayonet Neill–Concelman) to microdot wire (BCM-74-6W, Olympus, Center Valley, PA, USA). A commercial digital oscilloscope (model TBS1202B, Tektronix, CA, USA) was used to record the received pulse.

**Fig. 1:**
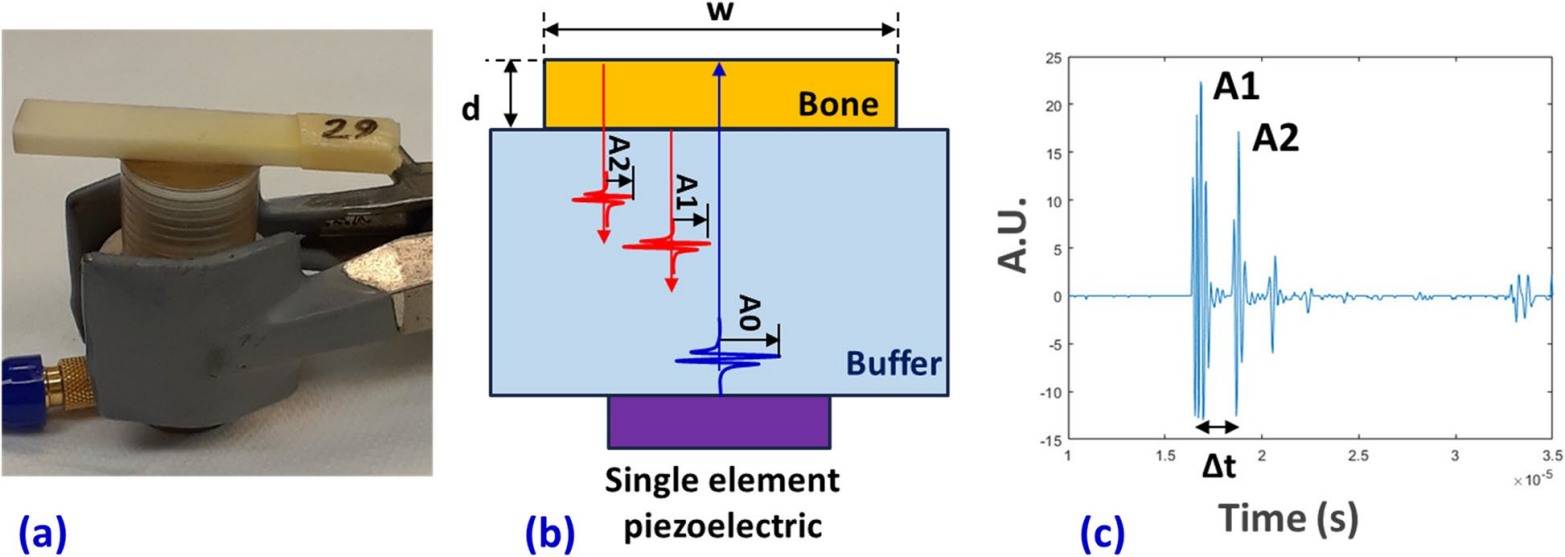
**a** Experimental setup for QUS measurement using a single-element transducer at a nominal 5-MHz center frequency (2.3 MHz actual center frequency). Bone specimens were placed on top of the transducer, and the wet surface provided an adequate acoustic coupling. **b** Schematics of the generated trigger pulse by the piezoelectric element (A0 amplitude) transmitting through the buffer and echoes reflected from the bone-buffer interface (A1 amplitude) and bone-air interface (A2 amplitude). Bone thickness and width are referred to as *d* and *w*, respectively. **c** A representative received an echo series in the time domain. The time of flight difference is referred to as Δ*t*

The specimens were kept hydrated by the operator using a dropper filled with phosphate-buffered saline during the five-minute QUS assessment. Each specimen was evaluated three times, and the average results were used for the correlational investigation. Figure 1a shows the experimental setup for the QUS measurement where bone specimens were placed on top of the transducer, and the wet surface provided a consistent acoustic coupling. As depicted in the scheme in Fig. 1b, the trigger pulse by the piezoelectric element (A0 amplitude) transmits through the polyethylene buffer rod, and reflected echoes are received from the bone-buffer interface (A1 amplitude) and bone-air interface (A2 amplitude). A representative received pulse echo series in the time domain is illustrated in Fig. 1c, where the amplitude of the first two echoes and the time of flight (TOF) differences are labeled as A1, A2, and Δt, respectively.

Equations 1 and 2 were used to measure SoS and *α* coefficients, respectively, where *R* refers to the reflection ratio in the bone-buffer interface. Details of the equation derivations are provided by Lees et al. [53]. *d* represents specimen thickness, measured using microcomputed tomography (μCT), as described below:

1 $$\textrm{SoS}\ \left[\textrm{km}/\textrm{s}\right]=\frac{2d}{\Delta t}$$

2 $$\left\{\begin{array}{c}R=\frac{A1}{A0}\\ {}\alpha\ \left[\textrm{dB}/\textrm{mm}\right]=20\times \frac{\log \left[{\left(1-R\right)}^2\times \frac{A0}{A2}\right]}{2d}\end{array}\right.$$

### UTE-MRI protocol

For MRI scans, specimens were placed in a plastic container filled with perfluoropolyether (Fomblin, Ausimont, NJ, USA) to minimize dehydration and susceptibility artifacts. The UTE-MRI scans were performed on a 3-T clinical scanner (MR750, General Electric Healthcare, WI, USA) using a single channel transmit/receive birdcage coil (BC-10, Mayo Clinic, MN, USA).

Absolute water proton density measurements (TWPD, BWPD, and PWPD) in bone strips were performed through signal comparison between bone and a reference rubber phantom of known proton density (equivalent to 22 mol/L H1, T2 ≈ 1.5 ms, T1 ≈ 250 ms). The required equations to calculate the proton densities are given in previous studies [11, 54]. The following imaging protocol was performed to estimate water proton densities:

- A proton-density (PD)-weighted 3D UTE sequence (repetition time [TR] 100 ms, echo time [TE] 0.032 ms, and flip angle [FA] 10°) for TWPD measurement
- A 3D inversion-recovery UTE sequence (TR 150 ms, TI 64 ms, TE 0.032 ms, and FA 20°) with a T1 BW of 135 ms for BWPD measurement [55]

PWPD was calculated by subtracting BWPD from TWPD.

For evaluation of macromolecular properties, a set of 3D UTE-MT sequences was performed with three different saturation pulse powers (400°, 600°, and 800°) at five different frequency offsets (2, 5, 10, 20, 50 kHz) (TR 100 ms, FA 7°). For UTE-MT modeling, the acquired data with the set of MT saturation pulse powers were fitted by a modified rectangular pulse approximation approach, previously described [56–58]. A Gaussian lineshape function was used to model the macromolecular proton spectrum and the loss of the longitudinal magnetization of the macromolecular pool [57]. Macromolecular fraction (MMF) and T2 (T2-MM) were the main two outcomes of the UTE-MT modeling. As a prerequisite for UTE-MT modeling, T1 measurement was performed using a UTE-based actual FA imaging-variable TR (UTE-AFI-VTR) sequence (AFI: TE 0.032 ms, TRs 20 ms and 100 ms; VTR: TE 0.032 ms, TRs 20, 40, 100, and 150 ms, FA 45°) [59]. T1 was measured based on a single-component exponential fitting (*S*(*TR*) ∝ 1 − exp(−*TR*/*T*1) + constant) of the acquired data [59]. The MT ratio (MTR) was defined as (MT_OFF_ - MT_ON_)/MT_OFF_ in percentage.

### Microcomputed tomography (μCT)

Bone strips were scanned using a μCT scanner (Skyscan 1076, Skyscan, Kontich, Belgium) at 9-μm isotropic voxel size. Specimens were scanned in the presence of two hydroxyapatite phantoms (0.25 and 0.5 g/cm^3^) for measuring BMD. Other scanning parameters were 100 kVp voltage, 100 mA current, 0.3° rotation step, and 5-frame averaging. A 0.05-mm aluminum and a 0.038-mm copper filter were used.

The μCT image segmentation was performed by gray-level thresholding. The gray level threshold was selected for each set of μCT data using the gray level histograms and visual investigation of the bone-pore interface in raw μCT images. Specimen thickness, width, and microstructural properties of each bone strip were calculated in a stack of slices covering 6 mm of the middle section of the strips’ length, corresponding to three MRI slices and the region placed on the US transducer.

Bone porosity was estimated as the ratio of the number of voxels in pores to the total number of voxels included in each bone strip. Pore size was also calculated as the diameter of the largest covering sphere within the pores [31]. Local BMD at each voxel was calculated using a linear function of the voxel’s gray level, which is determined based on the obtained gray levels of the two known BMD phantoms. Average pore size and BMD were calculated for each bone strip over the abovementioned 6-mm sections.

### Mechanical properties measurement

**Fig. 2:**
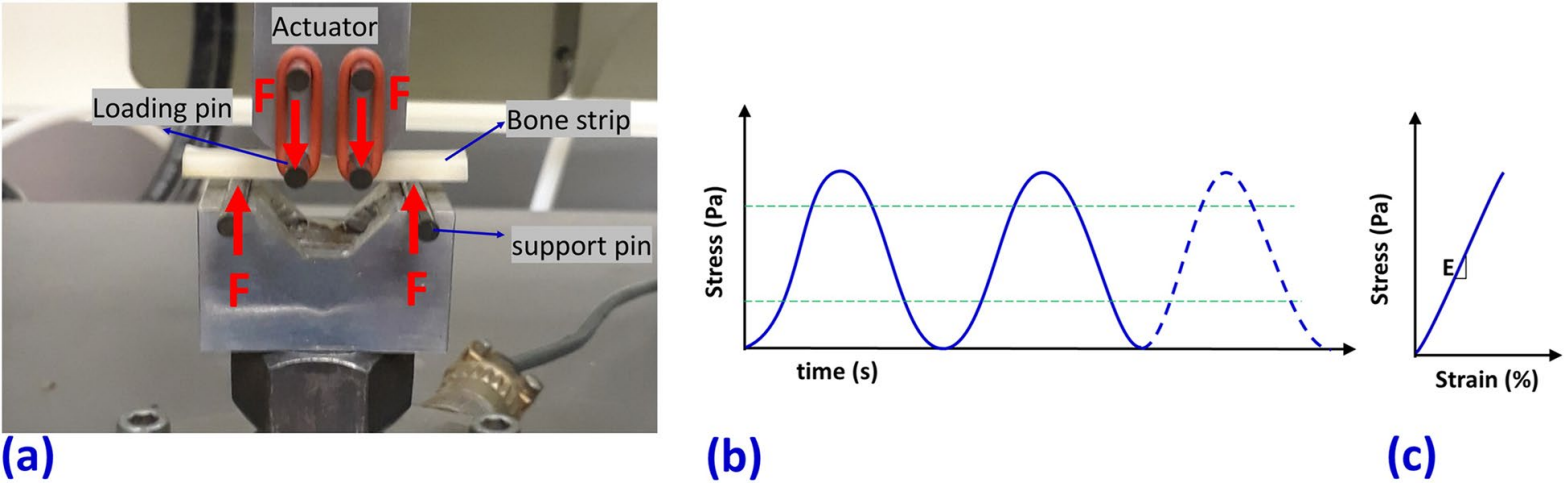
**a** Prepared bone strips mounted on the fabricated four-point bending jigs (aluminum holders and tungsten carbide pins) mounted on an Instron 8511.20 machine. The experiments were displacement-controlled at 10 Hz using a sinusoidal function with a maximum strain of 0.5% for 5 s. **b** Schematics stress-time curve shown for three loading cycles. **c** Schematics stress-strain curve which only the linear portion of it was acquired in this study for calculating Young’s modulus (E)

The dynamic tensile elastic modulus of each bone strip was measured using a 4-point bending setup [60]. The experimental setup is illustrated in Fig. 2a, which consists of four tungsten carbide pins (3-mm diameter) mounted on two aluminum holders. The upper holder was connected to the hydraulic actuator of a mechanical testing machine (model 8511.20, Instron, MA, USA). The lower aluminum holder was connected to a 4500-N load cell (model 41, Sensotec, OH, USA). Each bone strip was positioned on the lower pins. The contact between the loading pins and the bone strip was achieved by manually lowering the actuator. The dynamic mechanical test was performed below the yielding point according to the authors’ experience at 10 Hz using a sinusoidal function with a maximum strain of 0.5% for 5 s (Fig. 2b). The maximum measured force and displacement averaged for the 50 loading cycles (5 s at 10 Hz) were used to calculate the maximum stress (σ) and strain (ε) on the beam’s surface based on American Society for Testing and Materials standards. Young’s modulus of elasticity (E) was defined as the slope of the linear section of the stress-strain curve schematically demonstrated in Fig. 2c [60].

### Statistical analyses

The Kolmogorov-Smirnov test was used to examine the normality of the variable distributions. Since the variables were not normally distributed, Spearman’s rank correlations were calculated between the QUS, UTE-MRI, microstructural parameters (BMD, porosity, pore size), and Young’s modulus. Correlations with *p*-values below 0.05 were considered significant. Holm-Bonferroni method was used to correct the significance level for multiple comparisons. All measurements and models were performed using MATLAB (version 2021, The Mathworks Inc., MA, USA) codes developed in-house.

## Results

**Fig. 3:**
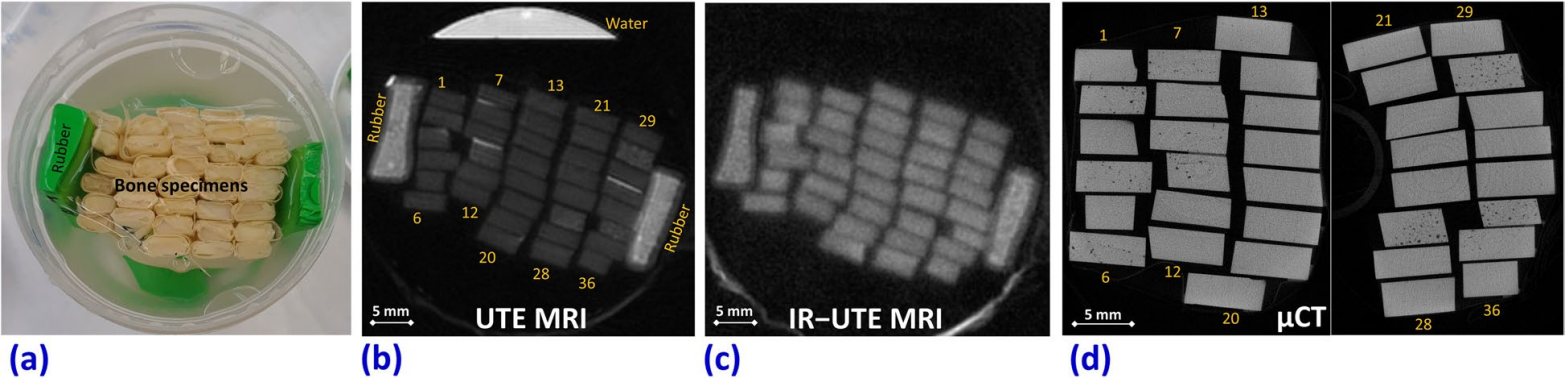
**a** Bone specimens (*n* = 36) placed in a plastic container filled with Fomblin. Ultrashort echo time magnetic resonance imaging (UTE MRI) (**b**) and inversion recovery UTE MRI (IR-UTE MRI) **c** images of the specimens in the axial plane (6 × 3 mm cross sections). **d** Microcomputed tomography (μCT) of the same cortical bone strips performed in two separate packages

Figure 3a shows the examined 36 bone strips and the rubber phantoms placed into a plastic container filled with Fomblin. Figure 3b, c shows the UTE-MRI and inversion-recovery UTE MRI images of the specimens in the axial plane, respectively, showing the 6 × 3 mm cross-sectional area of the specimens. The μCT images of the specimens are shown in Fig. 3d.

The median, interquartile, and total ranges of QUS, mechanics, μCT, and UTE-MRI measures of the bone strips are presented in Table 1. Spearman correlations of QUS-based measures (SoS and *α*) with mechanical (E), microstructural, and UTE-MRI-based measures are presented in Table 2. *α* demonstrated significant positive correlations with E (*R* = 0.69) and BMD (*R* = 0.44) while showing significant negative correlations with porosity (*R* = -0.41), T2-MM (*R* = -0.47), TWPD (*R* = -0.68), BWPD (*R* = -0.67), and PWPD (*R* = -0.45). SoS did not show significant correlations with any of the measures.

**Table 1:** Median, interquartile, and total ranges of QUS, mechanics, μCT, and UTE-MRI measures of the bone strips

QUS		Mechanics	μCT			UTE-MRI								
SoS (km/s)	*α* (db/mm)	E (GPa)	BMD (g/cm^3^)	Porosity (%)	Pore size (μm)	T1 (ms)	MMF (%)	T2-MM (μs)	TWPD (mol/L)	BWPD (mol/L)	PWPD (mol/L)	MTR-800 (%)	MTR-600 (%)	MTR-400 (%)
**3.20 ± 0.20 [2.70–3.40]**	7.30 ± 3.95 [0.70–12.40]	18.8 ± 2.4 [15.1–23.7]	1.30 ± 0.14 [1.10–1.46]	3.5 ± 4.3 [0.5–13.2]	71 ± 43 [43–147]	258 ± 10 [242–276]	34.8 ± 4.2 [29.2–38.5]	11.3 ± 0.3 [10.9–11.8]	11.2 ± 1.0 [10.0–14.6]	6.4 ± 0.85 [5.4–7.4]	4.8 ± 0.8 [4.2–8.0]	42.7 ± 2.5 [38.2–44.4]	31.3±1.6 [27.6–32.5]	17.1±1.2 [14.8–18.1]

*α* Attenuation coefficient, *BMD* Bone mineral density, *BWPD* Bound water proton density, *E* Elastic modulus, *μCT* Microcomputed tomography, *MMF* Macromolecular fraction, *MRI* Magnetic resonance imaging, *MTR* Magnetization transfer ratio (MTR-800, MTR-600, and MTR-400 refer to MTR at 2KHz for 800°, 600°, and 400° pulse power level), *PWPD* Pore water proton density, *QUS* Quantitative ultrasound, *SoS* Speed of sound, *T2-MM* Macromolecular T2, *TWPD* Total water proton density, *U*TE Ultrashort echo time

**Table 2:** Spearman correlation coefficients of QUS-based measures with mechanical, microstructural, and UTE-MRI-based properties of the cortical bone strips

	E	BMD	Porosity	Pore size	T1	MMF	T2-MM	TWPD	BWPD	PWPD	MTR-800	MTR-600	MTR-400
**SoS**	0.01 (*p* = 0.989)	0.23 (*p* = 0.177)	-0.18 (*p* = 0.295)	0.23 (*p* = 0.190)	0.07 (*p* = 0.671)	-0.19 (*p* = 0.278)	-0.23 (*p* = 0.193)	-0.14 (*p* = 0.419)	-0.34 (*p* = 0.046)	-0.03 (*p* = 0.862)	0.02 (*p* = 0.927)	0.09 (*p* = 0.602)	0.01 (*p* = 0.965)
***α***	**0.69 (*p*** **< 0.001)**	**0.44 (*p*** **= 0.008)**	-**0.41 (*p*** **= 0.010)**	-0.24 (*p* = 0.165)	-0.03 (*p* = 0.868)	0.20 (*p* = 0.258)	-**0.47 (*p*** **= 0.005)**	-**0.68 (*p*** **< 0.001)**	-**0.67 (*p*** **< 0.001)**	-**0.45 (*p*** **= 0.006)**	0.31 (*p* = 0.073)	0.28 (*p* = 0.109)	0.30 (*p* = 0.085)

*α* Attenuation coefficient, *BMD* Bone mineral density, *BWPD* Bound water proton density, *E* Elastic modulus, *μCT* Microcomputed tomography, *MMF* Macromolecular fraction, *MRI* Magnetic resonance imaging, *MTR* Magnetization transfer ratio (MTR-800, MTR-600, and MTR-400 refer to MTR at 2 kHz for 800°, 600°, and 400° pulse power level), *PWPD* Pore water proton density, *QUS* Quantitative ultrasound, *SoS* Speed of sound, *T2-MM* Macromolecular T2, *TWPD* Total water proton density, *U*TE Ultrashort echo time

Table 3 presents Spearman correlations of UTE-MRI-based measures with E, BMD, porosity, and pore size. E significantly correlated with TWPD, BWPD, and PWPD. BMD showed significant correlations with T1, TWPD, and PWPD. Porosity significantly correlated with T1, MMF, TWPD, PWPD, MTR-800, and MTR-600. Pore size significantly correlated with TWPD.

**Table 3:** Spearman correlation coefficients of UTE-MRI-based properties with mechanical and microstructural measures of the cortical bone strips

	*E*	BMD	Porosity	Pore size
**T1**	-0.41 (*p* = 0.014)	**0.46 (*p*** **= 0.006)**	**0.63 (*p*** **< 0.001)**	-0.06 (*p* = 0.774)
**MMF**	0.31 (*p* = 0.070)	-0.37 (*p* = 0.026)	-**0.50 (*p*** **= 0.002)**	0.31 (*p* = 0.150)
**T2-MM**	-0.39 (*p* = 0.022)	0.32 (*p* = 0.065)	0.18 (*p* = 0.287)	-0.17 (*p* = 0.435)
**TWPD**	-**0.62 (*p*** **< 0.001)**	**0.59 (*p*** **< 0.001)**	**0.42 (*p*** **= 0.013)**	-**0.57 (*p*** **= 0.004)**
**BWPD**	-**0.46 (*p*** **= 0.005)**	0.39 (*p* = 0.021)	-0.01 (*p* = 0.962)	-0.47 (*p* = 0.023)
**PWPD**	-**0.59 (*p*** **< 0.001)**	**0.61 (*p*** **< 0.001)**	**0.75 (*p*** **< 0.001)**	-0.47 (*p* = 0.023)
**MTR-800**	0.42 (*p* = 0.013)	-0.43 (*p* = 0.010)	-**0.56 (*p* = 0.001)**	0.41 (*p* = 0.054)
**MTR-600**	0.40 (*p* = 0.019)	-0.41 (*p* = 0.013)	-**0.50 (*p* = 0.002)**	0.42 (*p* = 0.048)
**MTR-400**	0.29 (*p* = 0.094)	-0.30 (*p* = 0.082)	-0.41 (*p* = 0.015)	0.57 (*p* = 0.005)

*BMD* Bone mineral density, *BWPD* Bound water proton density, *E* Elastic modulus, *μCT* Microcomputed tomography, *MMF* Macromolecular fraction, *MRI* Magnetic resonance imaging, *MTR* Magnetization transfer ratio (MTR-800, MTR-600, and MTR-400 refer to MTR at 2KHz for 800°, 600°, and 400° pulse power level), *PWPD* Pore water proton density, *T2-MM* Macromolecular T2, *TWPD* Total water proton density, *U*TE Ultrashort echo time

**Fig. 4:**
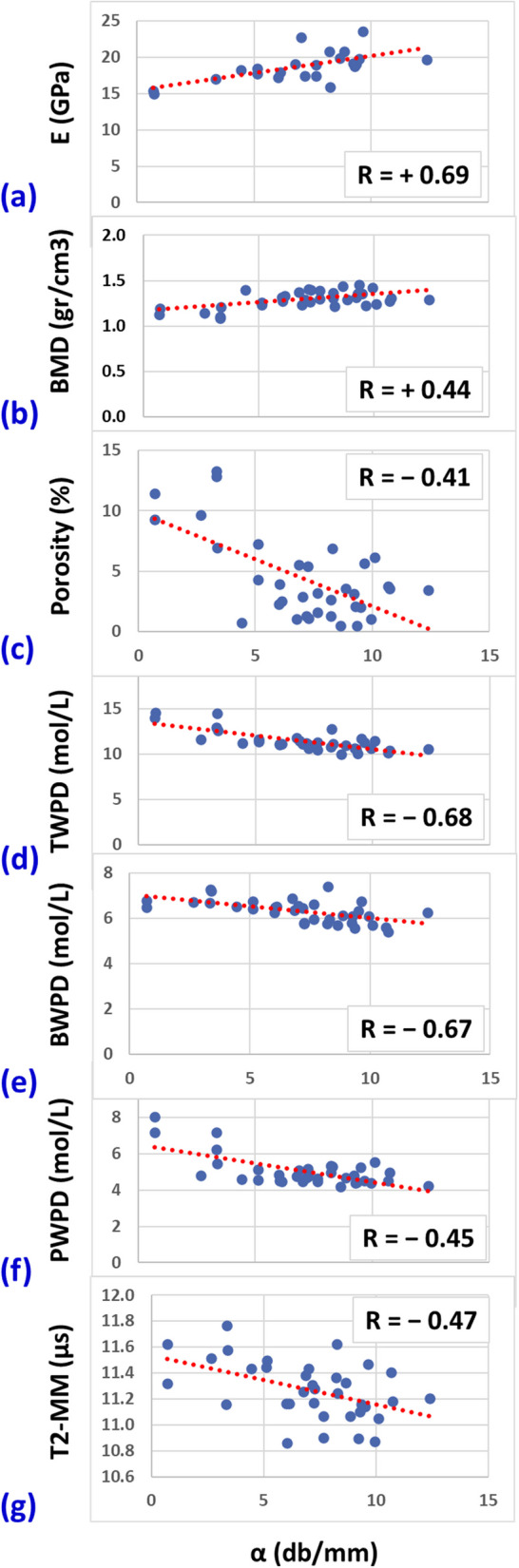
Scatterplots and linear trendlines of the *α* coefficient *versus* (**a**) elastic modulus (E), (**b**) bone mineral density (BMD), (**c**) porosity, (**d**) total water proton density (TWPD), (**e**) bound water proton density (BWPD), (**f**) pore water proton density (PWPD), and (**g**) macromolecular T2 (T2-MM). *R* values are Spearman correlation coefficients

The scatter plots and the linear trendlines of the *α* *versus* E, BMD, porosity, T2-MM, TWPD, BWPD, and PWPD are illustrated in Fig. 4, with significant correlations.

## Discussion

This study is the first to investigate the correlations of QUS measures with cortical bone water and macromolecular contents (estimated with UTE-MRI techniques) and with mechanical and microstructural parameters. The attenuation coefficient *α* demonstrated significant positive correlations with E and BMD while showing significant negative correlations with porosity, T2-MM, TWPD, BWPD, and PWPD. Remarkably, elastic modulus E showed higher correlations with *α* than with UTE-MRI measures. That is likely due to the mechanical nature of the US waves incorporated in the QUS assessment. Moreover, *α* correlations with BMD and porosity correlations were lower than correlations with water contents estimated by UTE-MRI.

The negative significant correlation between *α* and T2-MM is likely indicating the relationship between QUS and collagen matrix organization. Specifically, the mechanical properties of the bone collagenous matrix may add a source of US attenuation [61]. A shorter transverse relaxation time can be hypothesized to represent a higher order of organization of the fibers and a denser collagenous matrix. The significant negative *α*/PWPD and *α*/TWPD correlations agree with the positive correlations of *α* with BMD as more water is expected in regions with lower BMD in bone. Higher *α* correlations with BWPD than PWPD may indicate that water organized in finer structure (bound to matrix) provides lower acoustic impedance than water in larger pores. However, such conclusions require comprehensive investigations using ground truth histology, scanning electron microscope, and biochemical evaluations of bone specimens. Nevertheless, this study highlights the importance of future investigations exploring the relationship between QUS measures and all major components of the bone, particularly the collagenous matrix and water distributed as BW and PW.

The calculated attenuation coefficients in this study were in the range of the previously reported values employing transducers with nominal 5-MHz center frequencies [50, 53, 62]. The correlation between the attenuation coefficient and BMD in cortical bone has not been reported in the literature to the authors’ knowledge. In this study, we were not able to measure the frequency-dependent attenuation coefficient (slope of *α* against frequency, normalized broadband ultrasound attenuation [nBUA]). While nBUA is a commonly investigated QUS measure correlated with BMD in the literature, it is mostly examined in trabecular bones. The nBUA measurement often takes place using the US transverse transmission technique by comparing the received US signal with a reference phantom. The correlation between nBUA and BMD has been reported for bovine bone cortical bone in the radial direction using the US transverse transmission technique (*R* = -0.75) [50]. Such correlations were lower in axial and tangential directions (*R* = -0.62 and -0.66, respectively) [50]. Remarkably, correlations between nBUA and BMD in the literature were negative in cortical bone [50] while positive in trabecular bone [38, 50, 63]. Such controversial patterns were described due to the range of the BMD, such that for high BMD values in cortical bone and some trabecular bone specimens, the nBUA/BMD correlation may become negative [45, 50, 64].

SoS did not show significant correlations with mechanical, μCT- and MRI-based results in the current study. This was likely due to a very limited range of SoS in our specimens [2.70–3.40 km/s] as a result of similarly dense bone strips, which makes it difficult to draw any correlations. Our SoS measures were in the range of previously reported values between 3.0 and 4.5 km/s in cortical bone, depending on the experiment direction (radial, axial, or tangential) [45, 65–68]. On the other hand, the pulse-echo TOF method used in the current study is sensitive to specimen positioning, which might cause undesired experimental variations. Moreover, TOF measurement for SoS calculation may suffer from low reproducibility where detecting the same peaks in the triggered and reflected pulses is difficult.

Notably, previous investigations using US transmission techniques have reported significant correlations between SoS and BMD. For example, Yamato et al. reported a strong correlation between SoS in the axial direction of bovine cortical bone and BMD (*R* = 0.71) using the US transverse transmission technique at 10 MHz [68]. SoS correlation with BMD has been widely investigated in trabecular bone assessment, which resulted in slightly higher correlations with BMD (*R* = 0.66–0.87 in [45, 63]).

The UTE-MRI correlations with mechanical and microstructural properties in this study were slightly lower than those previously reported on human bone specimens [35, 51, 69–71]. This was likely due to the higher porosity and BMD ranges in previously investigated human specimens, which reduced the UTE-MRI sensitivity to compositional and ultrastructural differences in the specimens.

The accessibility and affordability of QUS techniques are attracting an increasing number of research groups to explore the capabilities of such techniques in bone assessment [36, 37]. Although different QUS methods have been developed and examined in the literature, the transverse transmission techniques have received more attention, potentially due to their simpler setup, and they are currently being translated into clinical trials [37, 41, 43, 46, 72]. Nevertheless, despite a few decades of research in QUS application for bone assessment, more validation and translational investigation are required to introduce such techniques to clinics for patient monitoring.

This study had a number of limitations. First, we performed the study on a number of bovine bone specimens with a low dynamic range of parameter values, which are likely different from the human bone ranges. The age and sex were not recorded, which might affect the final results of this study. To enable future *in vivo* studies, similar investigations should be performed on human bone specimens with larger sample sizes. Second, this study was performed *ex vivo* on bone specimens of unknown age cut from pure cortical bone layers. The presence of fat, muscles, and other soft tissues; a higher body temperature [33]; and subject motion will all contribute to differences in the performance of all QUS and UTE-MRI-based techniques *in vivo* compared with *ex vivo* studies. Third, exploring the QUS correlations with ground truth compositional and ultrastructural evaluations using histology or SEM would be advantageous in future studies. This connection would be crucial to postulate the role of QUS in clinical practice and to define its usefulness in evaluating human bone properties. Fourth, the QUS method in this study was based on pulse-echo and TOF methods that are repeatable, but corrections to the measurements might be required [73]. Investigating other QUS techniques, particularly the transverse transmission techniques currently translated into clinical trials [37, 41, 43, 46, 72], and their relationship with MRI-based compositional methods is an appropriate future study before promoting any specific QUS technique. Fifth, ultrasound generally suffers from lower reproducibility compared with other medical imaging modalities, which use volumetric acquisition, particularly for *vivo* applications. Although QUS methods are developed to improve the reproducibility of such measurements, future studies should be performed to investigate the reproducibility of these techniques in clinical settings.

In conclusion, correlations of QUS measures with cortical bone water and macromolecular contents and mechanical and microstructural parameters were investigated. The US attenuation demonstrated significant positive correlations with E and BMD while showing significant negative correlations with porosity, T2-MM, TWPD, BWPD, and PWPD. Remarkably, elastic modulus E showed higher correlations with *α* than with UTE-MRI measures. That is likely due to the mechanical nature of the US waves incorporated in the QUS assessment. The negative significant correlation between *α* and T2-MM is likely indicating the relationship between QUS and collagen matrix organization. This study highlights the importance of future investigations exploring the relationship between QUS measures and all major components of the bone, particularly the collagenous matrix and water distributed as BW and PW. This study is preliminary to further studies investigating more concretely the possible clinical application of QUS in cortical bone evaluation.

## Data Availability

The datasets used and/or analyzed during the current study are available from the corresponding author upon reasonable request.

## Abbreviations

*α*: Attenuation coefficient
μCT: Microcomputed tomography
3D: Three-dimensional
BMD: Bone mineral density
BW: Bound water
BWPD: Bound water proton density
DXA: Dual-energy x-ray absorptiometry
E: Elastic modulus
FA: Flip angle
MMF: Macromolecular fraction
MRI: Magnetic resonance imaging
MT: Magnetization transfer
MTR: Magnetization transfer ratio
nBUA: Normalized broadband ultrasound attenuation
PW: Pore water
PWPD: Pore water proton density
QUS: Quantitative ultrasound
SoS: Speed of sound
T2-MM: Macromolecular T2
TOF: Time of flight
TR: Repetition time
TW: Total water
TWPD: Total water proton density
US: Ultrasound
UTE: Ultrashort echo time
UTE-MT: Magnetization transfer ultrashort echo time
